# Supplementary figures and images for: A natural bacterial pathogen of C. elegans uses a small RNA to induce transgenerational inheritance of learned avoidance
Source: PLoS Genet. 2024 Mar 28;20(3):e1011178. doi: 10.1371/journal.pgen.1011178 (PMC10977744; doi:10.1371/journal.pgen.1011178)

■ 36 h OP50 trained in P0  
■ 36 h BIGb0170 trained in P0

P0 *Sphingobacterium multivorum*

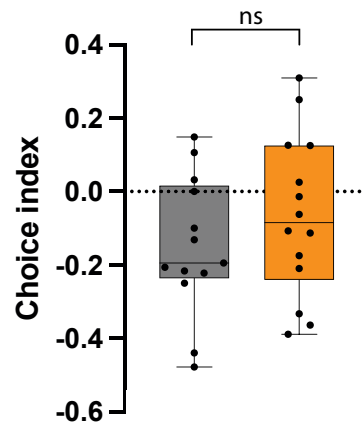

Supplement: S1 Fig — Each dot represents an individual choice assay plate. Boxplots: center line, median; box range, 25th–75th percentiles; whiskers denote minimum-maximum values; ns, not significant; Unpaired, two-tailed Student’s t test. (PDF) [file pgen.1011178.s001.pdf]

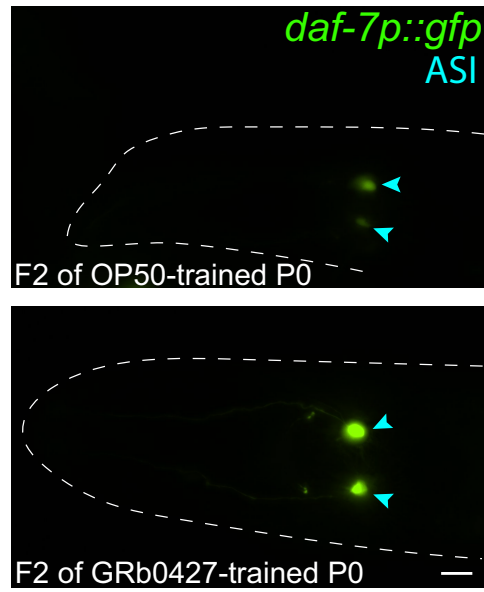

S2 Figure

Supplement: S2 Fig — Scale bar = 10 μm. (PDF) [file pgen.1011178.s002.pdf]

A

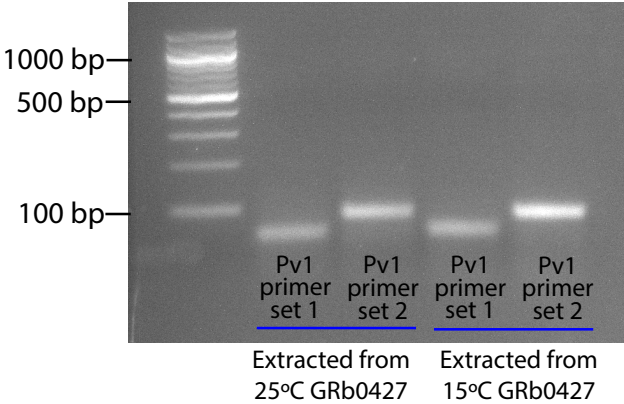

B

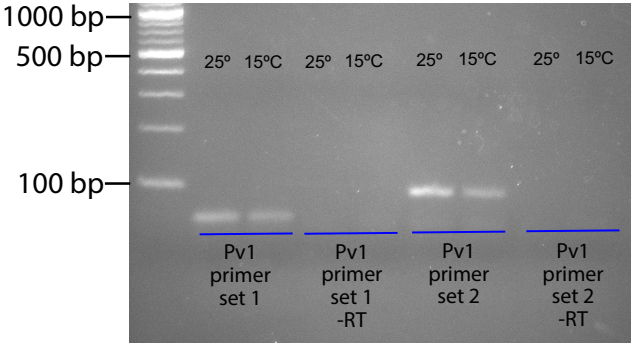

S3 Figure

Supplement: S3 Fig — (A) 62 bp and 88 bp of Pv1 amplified by two different primer sets (set 1 and set 2) from total RNA pool extracted from 25°C and 15°C-grown GRb0427 (indicating expression of the Pv1 small RNA under both temperature conditions). (B) 62 bp and 88 bp of Pv1 amplified by the same primer sets as in (A) (also see Methods) from small RNA pool extracted from 25°C and 15°C-grown GRb0427. +RT and -RT indicate presence and absence respectively of the reverse transcriptase enzyme. (PDF) [file pgen.1011178.s003.pdf]

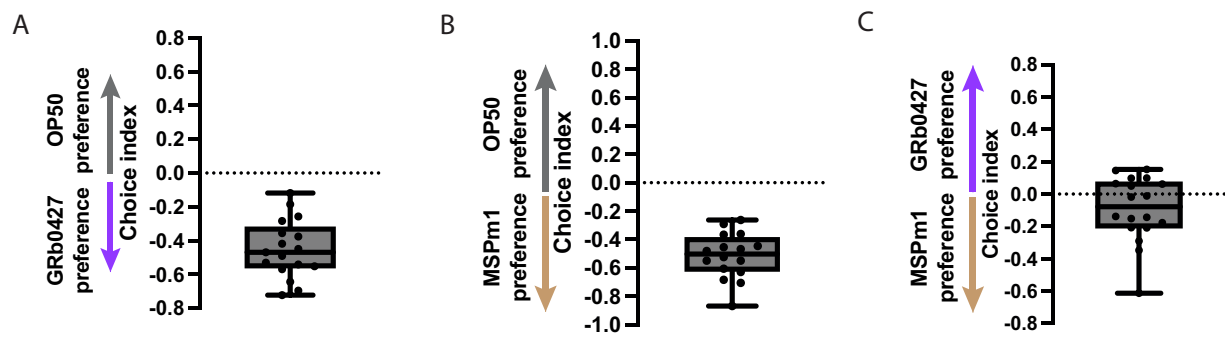

S4 Figure

Supplement: S4 Fig — (A-C) Naïve worms do not exhibit a preference towards either P. vranovensis GRb0427 or P. mendocina MSPm1 in a GRb0427-MSPm1 choice assay (A) while showing preference for GRb0427 and MSPm1 with respect to OP50 (B,C). (PDF) [file pgen.1011178.s004.pdf]
