# Supplementary material for: A natural bacterial pathogen of C. elegans uses a small RNA to induce transgenerational inheritance of learned avoidance
Source: PLoS Genet. 2024 Mar 28;20(3):e1011178. doi: 10.1371/journal.pgen.1011178 (PMC10977744; doi:10.1371/journal.pgen.1011178)
Supplement: Attachment — Submitted filename: GRb response CTM_013124.pdf [file pgen.1011178.s006.pdf]

New experiments we have added to the paper to address all of the reviewers' concerns (and extra):

1. *sid-1* – shows that dsRNA transport is required for GRb avoidance, just as it is for PA14 avoidance
2. *dcr-1*– shows that Dicer function is required for GRb avoidance, just as it is for PA14 avoidance
3. *sid-2*– shows that environmental dsRNA transport is required for GRb avoidance, just as it is for PA14 avoidance
4. *hrde-1*– shows that the 22G RNA binding Argonaute is required for GRb avoidance, just as it is for PA14 avoidance
5. *maco-1* qPCR: F2, F4, and F5 generations – shows that *maco-1* indeed is regulated by GRb/Pv1 sRNA treatment in P0-F4
6. Horizontal transfer of GRb memory – this works just as it did for PA14/P11, and Cer1 is required
7. additional *Pdaf-7::gfp* counts on Grb and PA14
8. *Pdaf-7::gfp* imaging in F2, F4 and F5 progeny following GRb training.
9. *Pdaf-7::gfp* on GRbΔ16 – shows that there is no response if the perfect match in Pv1 is removed
10. Wild strains w/wild bacteria JU1580
    a. JU1580 learned avoidance after GRb training
    b. P0-F4 avoidance after GRb training
    c. P0-F4 avoidance after GRb sRNA training
    d. GRb avoidance after training on the Intergenic region (IntReg) containing the *maco-1* match
11. ED4030 learned avoidance after training on GRb
12. Controls for preferences (GRb vs OP50)
13. Additional negative data – longer (36hr) training does not induce avoidance of *S. multivorum*
14. Preference between GRb and GRbΔ16
15. New 4nt mismatch Pv1 sRNA shows that when four of the nucleotides within the loop of the 16nt *maco-1* match are changed, the sRNA no longer induces avoidance.

We also added a model figure to include the new genetic data.

Reviewer #1: C. elegans exhibits a remarkable tendency for transgenerational epigenetic inheritance of RNAi-mediated silencing. Whilst mechanistically this is fairly well characterised, the ecological context of this system, if indeed it exists, is largely unknown. In the current manuscript the authors build on several previous papers from the same team in which they purport to explain transgenerational RNAi may be an evolved response to pathogenic bacteria encountered by C. elegans. Previously their model was based on

experiments with P. aeruginosa, which is not a natural pathogen of C. elegans. In the current manuscript they argue that a natural pathogen of C. elegans, P. vavroensis (previously described) has the same effect.
The manuscript presents two claims which, I believe, the authors argue are important conceptual advances. First, they argue that the fact that P. vavroensis is a "natural" pathogen of C. elegans supports the ecological role of the transgenerational epigenetic inheritance that they observe in the laboratory. Second, they argue that, because P. vavroensis exposure can lead to C. elegans avoiding non-pathogenic food sources, their experiments "explain" why the transgenerational epigenetic inheritance has evolved to only last a limited number of generations. They also present a mechanism whereby the bacteria can induce a behavioural response that is inherited transgenerationally. If supported by strong data these advances would indeed be important.

Thank you for recognizing the importance of these conceptual advances.

However, I don't think that the experimental results support the strong claims made in the manuscript. Whilst the gaps in the mechanistic understanding could be filled by experiments (and I suggest some of the ways in which this could be done), the way in which the manuscript attempts to link their laboratory observations to ecological scenarios must be toned down or removed as they are extremely speculative and could never be justified by the kind of laboratory experiments that the authors put forward. Below, I outline these major issues in more detail.
Major issues
1. Mechanism
The authors suggest that a small non-coding RNA produced by the bacteria (i) is taken up by the worms (ii), processed by the RNAi pathway (iii) and triggers silencing of a specific genes maco-1 (iv) leading to the behavioural response that is inherited for 4 generations (v). More evidence is required for each of these steps.
i) The authors present no solid evidence that a small non-coding RNA is actually produced. They claim to have done small RNA sequencing of the bacteria but then just present (in Fig 7A) a structural model of a small RNA that they claim to have discovered. What is the evidence for this? At a minimum there needs to be some presentation of the genome-wide small RNA sequencing data from the bacteria, showing that small RNA is recovered from this region far above other non-coding regions of the genome, and that the start and end points of this match their predicted structure. Moreover, it would be

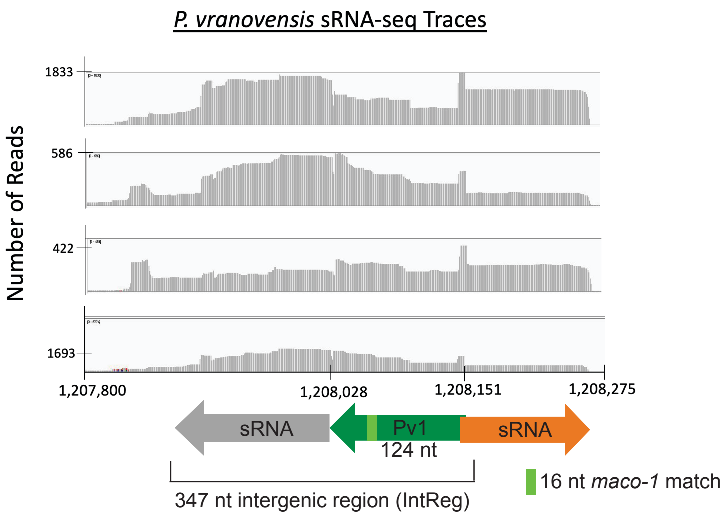

important to verify that this specific, highly structured, prediction actually corresponds to the RNA that is produced- some kind of RACE perhaps where they can map the 5' and 3' terminus of the RNA produced by the bacteria.

We are happy to provide our sRNA-seq results (NCBI BioProject #SUB13357277). Here you can see that we have added our results of multiple sequencing replicates in greater detail in the area surrounding the 16nt match to *maco-1* (see figure). These data show the boundaries of the Pv1 small RNA as well as its neighbors. As we show here, the Pv1 sRNA is within the region we identified as having the 16nt match to *maco-1*.

Ii/iii) There is no small RNA sequencing data of worms that have eaten the bacteria presented, so there's no evidence that the non-coding RNA produced by the bacteria is actually processed by the RNAi pathway (i.e. does it get chopped up by dicer). The authors should perform this experiment to show that short dsRNA really are produced from this sequence (see also point iii). Furthermore, the authors should perform the experiment in a sid-2 mutant as this would not be able to take up dsRNA from the bacteria and so, if the authors are correct, would not trigger the inheritance response.

Thank you for these suggestions. We have now tested the *sid-2(qt42), dcr-1(tm1200),* and *sid-1(qt9)* mutants on Pv1 sRNA, and we find that these three genes are all indeed required for Pv1-mediated avoidance learning, confirming that uptake and processing of bacterial sRNAs are required. This is the same mechanism we had previously shown for PA14 and P11 sRNA, and had proposed for *P. vranovensis* and Pv1 sRNA, supporting our model.

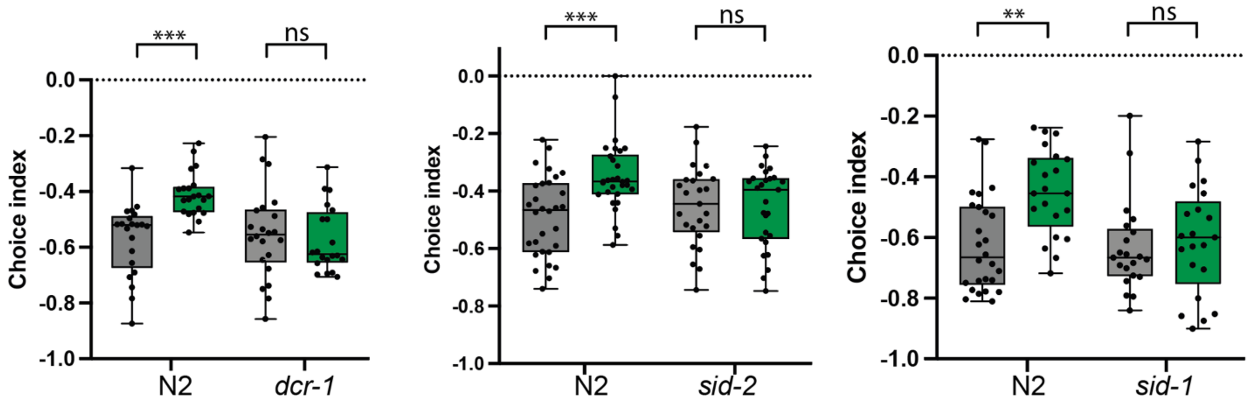

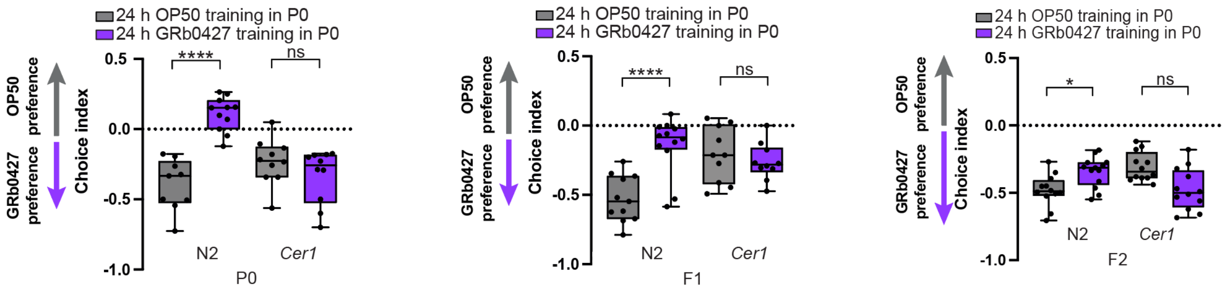


Although the reviewer did not request it, we have also added new data showing that the basic mechanism, including transgenerational inheritance of learned avoidance of GRb/ *P.vranovensis* (P0, F1, and F2) and horizontal transfer of information via Cer1 vesicles in conditioned media, is shared with the PA14 mechanism.

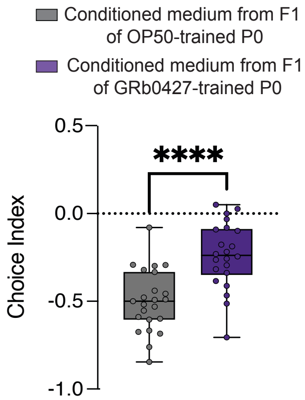


iv) For silencing to occur 22G-RNAs need to be induced against the maco-1 gene as a result of the limited sequence identity (16bp) in the non-coding bacterial RNA. I find this very hard to believe – if 16bp were sufficient to trigger silencing then C. elegans RNAI by feeding would be riddled with off-target effects and would be impossible to interpret. In RNAi by feeding much longer regions of sequence similarity ~100-200bp as a minimum are required. Therefore, the authors need to verify that they really are seeing maco-1 22G-RNAs triggered by feeding with P. vavroensis and the 347bp region containing the supposed non-coding RNA. The sequencing described in ii) would be able to recover these if the authors use RppH to remove the 5' triphosphate on 22G-RNAs.

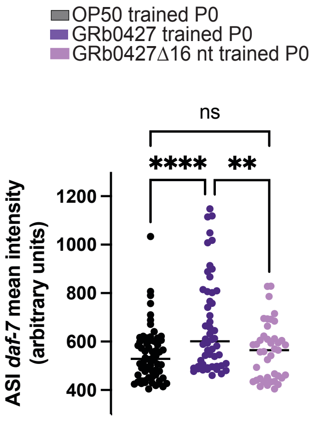


Please note that we are *not* suggesting that *only* 16nt of sequence would be sufficient for the effects we see, but rather that the *16nt in the context of the bacterial sRNA* is required. We have shown that the 16 nt region within the Pv1 sRNA is necessary for function (see behavior, Fig. 7f) and for *Pdaf-7::gfp* expression changes (new *daf-7::gfp* data, right), and that GRb knocks down *maco-1*.

Furthermore, we have also engineered a set of mismatches in the Pv1 sRNA in the 16nt stem-loop *maco-1* match (while maintaining the stem-loop structure); this sRNA is unable to induce avoidance, unlike the normal Pv1, further supporting our model that the 16nt of perfect match to *maco-1* is absolutely necessary for the avoidance mechanism:

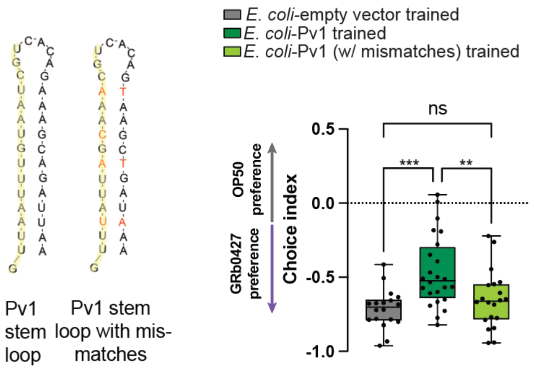


We have now also shown that *hrde-1* is also required for GRb's effects through the Pv1 sRNA. HRDE-1 is the nuclear Argonaute that binds to 22G RNAs, thus the requirement for *hrde-1* in Pv1-mediated learned avoidance suggests that these must be involved in the mechanism.

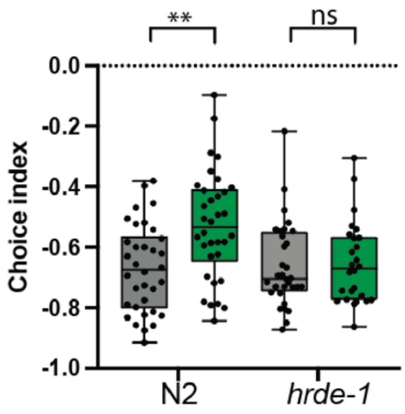


Together, our data suggest that the mechanism that we had previously identified for PA14-P11 sRNA processing is shared with *P. vranovensis*-Pv1 processing, and involves bacterial sRNA uptake (*sid-2*), sRNA processing (*dcr-1, hrde-1*), sRNA transport (*sid-1*), subsequent *maco-1* knockdown, then *daf-7* expression increase, and then behavioral switching from attraction to avoidance. Therefore, the simplest model is that GRb's small RNAs, including Pv1, are processed by the same mechanisms we have already shown for PA14's sRNAs, but do so through a different sRNA (Pv1) that specifically targets a different exon of *maco-1*. (Please note that previously we had shown that P11-mediated avoidance requires *sid-2, dcr-1, sid-1, daf-7, maco-1*, and also other components of the RNAi pathway, including *rrf-1, rrf-3, rde-1, rde-2, rde-4, mut-7, nrde-3, nrde-4, rde-3,* and *rde-8,* as well as components of the COMPASS complex, including *set-2, set-32, rbr-2, wdr-5.1*, and the piRNA regulators *prg-1*; it is not clear to us why we should re-test all of these components with Pv1, given that we understand what the difference between P11 and Pv1 is, we have shown that the target is another exon of *maco-1*, we know it affects *daf-7* expression, and here we have additionally shown the involvement of *sid-1, sid-2,* and *dcr-1* once more.)

*NB:* The studies the reviewer cites are from non-physiological conditions – i.e., artificial RNAi constructs expressed in foreign bacteria to knock down foreign GFP expression - that likely lack many aspects of the natural context, such as secondary and tertiary structure of sRNAs that are necessary for bacterial sRNA processing in *C. elegans*. Future directions for our lab include extensive biochemical experiments such as those 22G isolation experiments suggested, but are beyond the scope of the current work, which is focused on the genetics and ecological relevance of transgenerational epigenetic inheritance of learned avoidance.

Identifying the conserved features such as those we have uncovered here with Pv1 sRNA from *P. vranovensis* is a critical first step in understanding the "rules" of the sRNA-induced avoidance, which is why the current findings are so important.

v) P. vavroensis-induced gene expression changes should, if the authors' model is correct, last for 4 generations. There is no data presented to support whether maco-1 is altered in expression for 4 generations, F1, F2, F3, f4- qPCR in Fig 5E is only in P0- and then goes back to normal. This data is important and if the result of this shows that maco-1 does not show sustained downregulation, the authors model to explain their findings would be seriously undermined. The authors also mention that an independent dataset (Burton et al., 2020) showed downregulation of maco-1 but they do not specify whether this is in P0, F1 or F2 animals- this data is all in the cited dataset so they should quote figures for all of these generational time points. Again, it would strongly undermine their claims if the effect is only in P0.

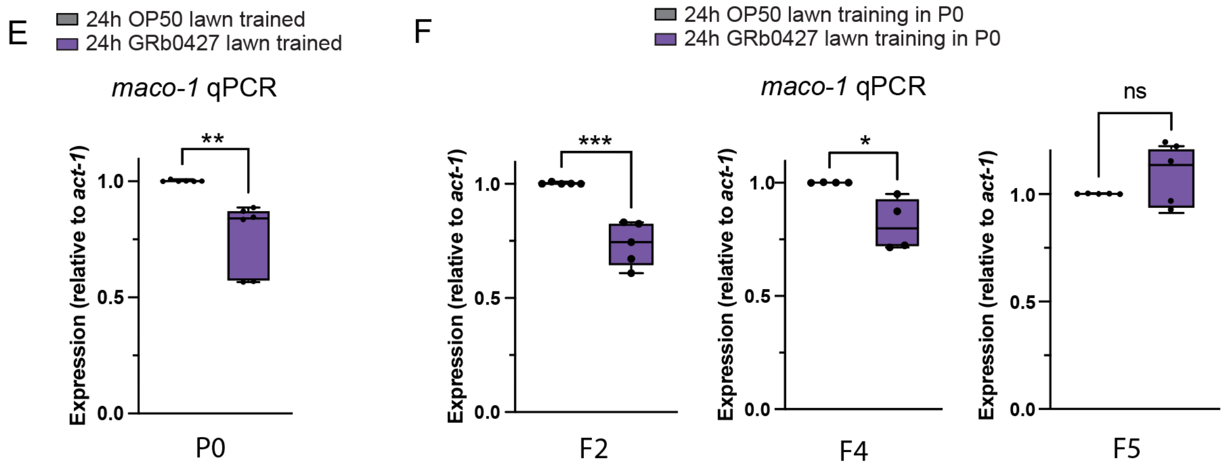


Our new measurements of *maco-1* by qPCR show that *maco-1* is downregulated not just in the P0, as we already showed, but also in F2 and F4, but then returns to the same levels as OP50-control treated animals in the F5.

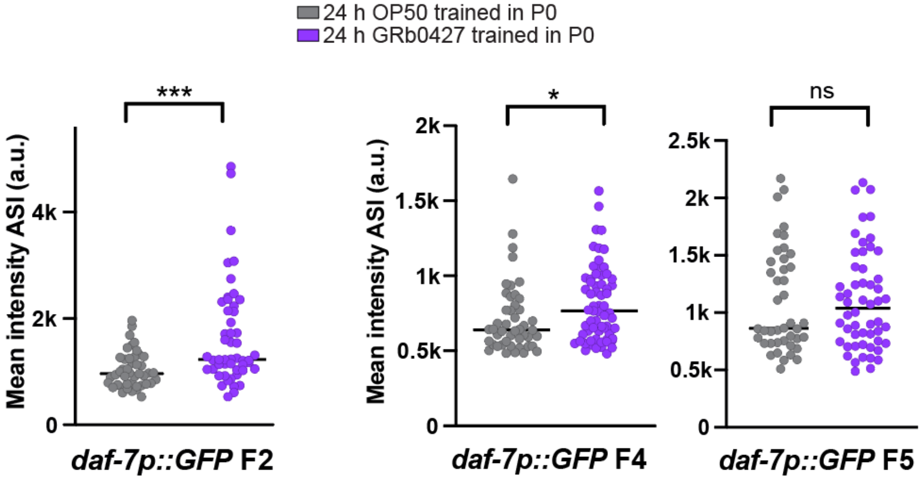


We should be clear that the sRNA-based mechanism of

*maco-1* downregulation and *daf-7* upregulation is not *only* a transgenerational response, but it is consistent with the avoidance response that is seen in P0-F4 that is mediated by the sRNA-based mechanism. It is critical that the reviewer and readers understand this point, and we will clarify this notion in the text. *Maco-1* downregulation, like *daf-7* upregulation in the ASI, is first observed in the P0 generation after PA14, *P. vranovensis*, bacterial sRNA, P11, or Pv1 training, because the mechanism that reduces *maco-1* happens via sRNAs in the P0. Like other aspects of the sRNA-based avoidance mechanism, if we see it in P0, we see it through F4.

We previously showed that *daf-7* expression is downstream of *maco-1* in P0 as well as later generations (Kaletsky et al. 2020) – that is, *daf-7* levels are regulated by *maco-1,* and *daf-7* expression does not increase upon P11 sRNA exposure in *maco-1* mutants - and that increased *daf-7* expression lasts through the F4 (Moore et al. 2019). Therefore, increased *daf-7* levels are an indicator of reduced *maco-1* levels.

In support of a similar mechanism at work in the processing of Pv1 sRNA from GRb/*P. vranovensis*, we have shown that:

1. like P11-induced PA14 avoidance, avoidance of GRb/*P. vranovensis* is transgenerational, lasting through the F4;
2. similarly, GRb/*P. vranovensis* sRNA induces avoidance through F4;
3. GRb/*P. vranovensis* and its sRNA induce *daf-7* expression transgenerationally;
4. the region expressing the Pv1 sRNA (IntReg) induces avoidance from P0 through F4;
5. training on the Pv1 sRNA induces avoidance transgenerationally;
6. *maco-1* mutants exhibit high naïve avoidance of GRb/*P. vranovensis*;
7. *maco-1* RNAi treatment induces high naïve avoidance of GRb/*P. vranovensis*;
8. *maco-1* is required for the ability to learn avoidance upon GRb/*P. vranovensis* training; and
9. GRb/*P. vranovensis* treatment reduces *maco-1* expression. (It should be noted that sRNA-mediated *maco-1* downregulation occurs in P0 as well as later generations, it is not only a transgenerational phenotype.)

Therefore, our experimental results support a model in which sRNA is taken up and processed through the RNAi and 22G pathway (*sid-2, dcr-1, sid-1, hrde-1*), resulting in downregulation of *maco-1* and subsequent upregulation of *daf-7,* causing in a switch in attraction to avoidance. In response to the reviewer's comments, we have added new *daf-7* and *maco-1* data, but these new results do not change the model. It is not clear, given all of the data we have shown, why the *maco-1* response would only be seen in P0, when the behavior and *daf-7* expression changes downstream of *maco-1* persist through F4.

Re. Burton et al., 2021 data: The sRNA-mediated avoidance phenotype is observed in *adult* P0-F4 animals (not just transgenerationally) - so we have no expectations about embryonic data. In fact, we do not expect to see differences in embryos, as we previously showed that the avoidance phenotype requires an adult germline. The Burton dataset only reported *adult* sequencing data for the P0 generation; they do not report adult data for any other generations. As a result, they may have missed any gene expression changes that manifest post-embryonically. In agreement with all of our other data, we observed that *maco-1* is downregulated in the adult sequencing data they present. We have added a note about the adult expression changes of *maco-1*. Thank you for this question.

2. Ecological relevance

The main reason why this study would be a substantial advance over the authors' previously published work on this topic would be that this is an ecologically relevant interaction. However, there is insufficient data to conclude this point. All we know is that P. vavroensis can be found with C. elegans in the wild and that, in the lab, it is pathogenic. We do not know that it is a pathogen in the wild and, to my knowledge, P. vavroensis has only been sampled once so we have no knowledge of how frequently C. elegans is likely to be exposed to it. Unless the authors can provide some evidence that this is a frequently observed interaction, their claim that this is somehow an evolved response is too strong and should either be toned down or removed. This then challenges the novelty of their observations since they have already published that Pseudomonas derived small RNAs from other species have an effect on behaviour, inherited transgenerationally, in the lab.

In the absence of an example of a study in which a group takes isolated *C. elegans* microbiome bacteria from the wild, shows a phenotype in the lab, genetically characterizes this phenotype, and then goes back to the wild to test behavior, we have further expanded upon our experimental results by testing wild *C. elegans* strains on GRb0427/*P. vranovensis* – that is, testing wild worm strains with wild bacterial strains. As shown here, we find that the wild

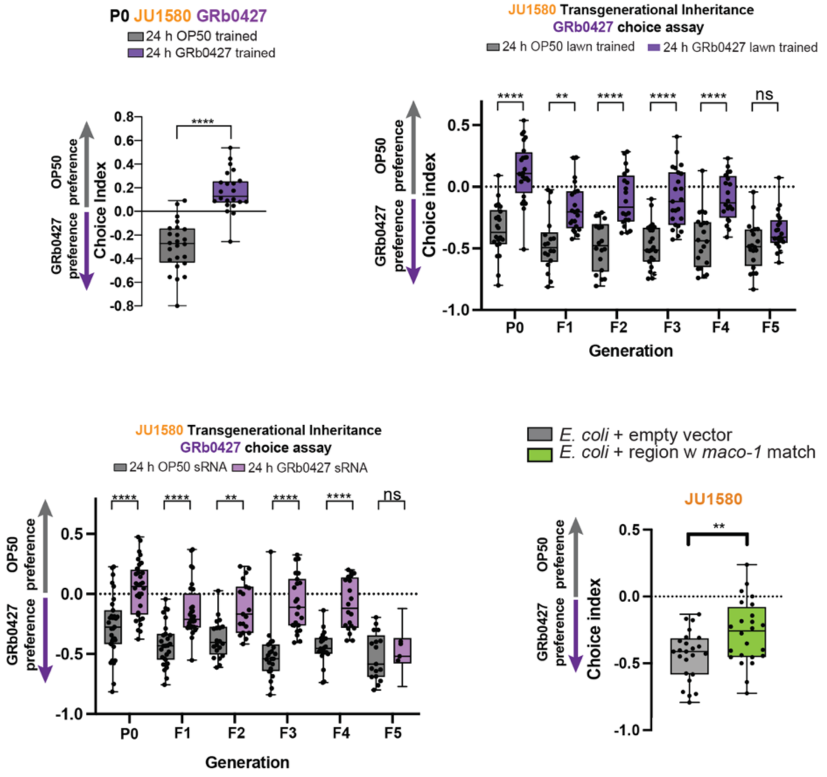

JU1580 worms (Felix et al. 2011), like N2, are first attracted to GRb0427/*P. vranovensis,* then learn to avoid it, and do so through F4 before returning to naïve avoidance in the F5 (two replicates shown). Furthermore, JU1580 does so through the same region containing Pv1 (green).

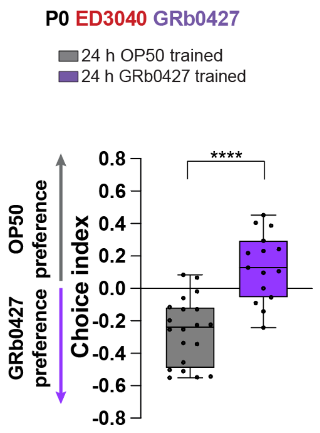


We have also tested ED3040, another wild worm strain (Dolgin et al. 2007) that has been tested for its interactions with the BIGbiome model microbiome (Zhang et al. 2021); ED3040 shows a similar attraction to and then learned avoidance of GRb/*P. vranovensis*. Therefore, it appears that wild worm strains behave like N2 in all of their interactions with this bacterial strain that was obtained in the wild *C. elegans* environment.

3. Rationale for limited transgenerational inheritance

The authors make the startling claim that the avoidance response may be limited because of the possibility that beneficial bacteria are avoided incorrectly. This goes way beyond what they actually show in the manuscript. Their experiment shows only that the exposure to pathogen triggers avoidance of a non-pathogenic bacteria as well but they do not show that there is a fitness cost associated with this even in the lab, let alone the wild. It would be possible to test this in the lab: a competition experiment between inheritance competent and inheritance incompetent strains (i.e. a WT vs a hrde-1 mutant) grown on a mixture of pathogenic and non-pathogenic bacteria or a fluctuating environment (pathogenic vs non-pathogenic in different generations). See which strain wins- if the inheritance incompetent strain wins then there may be some support for the authors' assertion. Even this, though, would not really be sufficient to address whether this is actually relevant in the wild. Thus I think this entire section (Fig 8) should be toned down to report simply what the experiment shows, which does not involve anything about the importance of "forgetting" the avoidance response, only that the response itself has some cross-species effects that might not be directly useful.

Thank you for your comments. We have moved our more speculative comments to the Discussion. However, we still need to explain the motivation and logic behind our experiments; specifically, since there is no difference in response at different temperatures, as we found for PA14, we needed to understand when there would ever be any pressure to lose the learned avoidance of *P. vranovensis*. This was the motivation behind testing whether training on *P. vranovensis* also induces avoidance of beneficial bacteria, which indeed turned out to be the case.

(We note that the reviewer's suggested experiment would not work, as the "inheritance incompetent" strain would not avoid the pathogen, and therefore then die on the pathogen in an early generation - not "win," as the reviewer has suggested.)

We previously showed that inheritance of the avoidance learning behavior benefits offspring because they avoid pathogen (Moore et al. 2019), which we can remind the readers of in the text; the question here is why is such avoidance not hard-wired or innate, and we suggest that avoidance of similar-smelling beneficial bacteria that are not pathogenic would eventually result in them unnecessarily avoiding beneficial food sources. We show that the worms cannot tell the difference between pathogenic (*P. vranovensis*) and beneficial (*P. mendocina*) strains after training, thus providing experimental evidence to support our hypothesis.

Reviewer #2: The manuscript by Sengupta et al builds on their previous work showing that the PA14 small RNA of the human pathogen Pseudomonas aeruginosa is required for transgenerational inheritance of learned pathogenic avoidance in C. elegans. In this current study, authors seek to determine if a similar mechanism operates in the worm's naturally occurring microbial environment. Authors survey nine bacterial species for the ability to induce learned avoidance and find one pathogenic species – Pseudomonas vranovenis/GRb0427 – that upregulates the daf-7p::gfp reporter in ASI neurons, similar to PA14. Unlike PA14, GRb0427 does not activate daf-7 expression in another set of neurons and does not robustly activate the innate immune response, suggesting a shared and distinct mechanism. The GRb0427 avoidance response is transmitted for four generations and requires a small RNA produced by GRb0427. Authors use a clever and rigorous strategy to identify the intergenic region Pv1 RNA, which has a 16-nt match to the maco-1 genes. (Whilst Pv1 and PA14 both have homology to maco-1, the matches are in different exons.) Pv1 and these 16nt are sufficient and necessary for transgenerational inheritance. Authors look at other Pseudomonas species and find that Pv1 induces avoidance of the beneficial bacterial MSPm1 and propose that "forgetting" by the 5th generation may protect C. elegans from maladaptive avoidance. These findings are exciting and important – this work and Kaletsky et al (2020) indicate that small RNA-mediated transgenerational inheritance may be a broad mechanism that C. elegans uses to avoid Pseudomonas pathogens. This manuscript is exceptionally well written, the figures are convincing and easy to follow, and the sophisticated genetic/genomic analysis combined are perfect for the readership of PLoS Genetics.

Thank you, we appreciate your comments!

I really have no criticisms or concerns. My only suggestion is to rearrange Figure 1 so that panels E, G, and D are directly above panels L, M, and N – this will make it easier to compare P0 with the F1 generation.

Thank you, this is a very good suggestion. (We have done our best to address the suggestions of order and labeling by Rev. 3 at the same time.) We have also made the ns/*** notations more obvious to help the readers.

Reviewer #3: In the manuscript “A natural bacterial pathogen of C. elegans uses small RNA to induce transgenerational inheritance of learned avoidance” Sengupta T et al perform a series of thorough experiments to investigate whether bacteria that C. elegans are naturally exposed to elicit the same transgenerational memory that they had previously demonstrated for an alternative bacteria that is pathogenic to humans (Pseudomonas aeruginosa). They showed that another Pseudomonas strain, Pseudomonas vranovensis, also elicits an epigenetic memory. They show this is dependent on the small RNAs isolated from the P. vranovensis. Furthermore they demonstrate that a specific small RNA which targets the same gene in C. elegans, maco-1, which they had demonstrated was targeted by P. aeruginosa small RNAs to elicit epigenetic memory, is also at play in the second Pseudomonas strain. They demonstrate that this small RNA is necessary and sufficient to induce the epigenetic memory by knocking it out in the Pseudomonas and expressing it in other types of bacteria. All in all I thought this is a very thorough and well done paper and mostly minor corrections are required for publication.

Major points

1) Are there beneficial memories with regards to the good bacteria? It seems like all the types of bacteria here are inducing an avoidance (even the beneficial one (Myb71)). Why are they learning to avoid the beneficial bacteria (Myb71) the same as they are the detrimental bacteria (GRb0427) in the P0 generation? This just seems like avoidance of foreign rather than avoidance of detrimental. Unless some beneficial memory can be demonstrated or if already published referenced then a restructuring of the framing of the manuscript is necessary. Can the small RNA (IntReg or Pv1) be added to a bacteria that produces beneficial memories (not just preferential bacteria) and change it to a negative multigenerational memory?

Thank you for your suggestion – we will rearrange the bacteria to group them into beneficial/neutral and pathogenic. We will also remind the readers that it was previously shown that worms learn to avoid the pathogen *S. marcesans* (Zhang et al. 2005) but only for one generation (Moore et al. 2019).

However, it should be noted that most (6 of the 9) bacteria we tested do NOT induce avoidance in the P0 generation (every bacterial species that has “ns” between the gray and colored bars) - that is why we continued to study only the remaining three bacterial strains for effects in the F1 generation. Two of these three had no effect in the F1, including Myb71; therefore, Myb71 was not the subject of further study or speculation. P0 avoidance of the beneficial Myb71 has already been reported (Petersen et al. 2021); those authors discussed but did not resolve why *C. elegans* avoids Myb71 after exposure, but the reviewer might be

interested in their study to address this question (they focused on colonization of the worm gut). Of the three bacteria tested, only one, *P. vranovensis*, induced intergenerational avoidance, which is why we tested it for transgenerational (F2) avoidance and focused on it for the rest of the work.

Your comment made us realize that our "ns" vs asterisk designation was not clear enough in our figures, nor was the grouping as logical as it should be, so we have made that clearer and rearranged the panels as Rev. 2 suggested. We have also restated this important observation in the text. We apologize for not making this foundational point clearer.

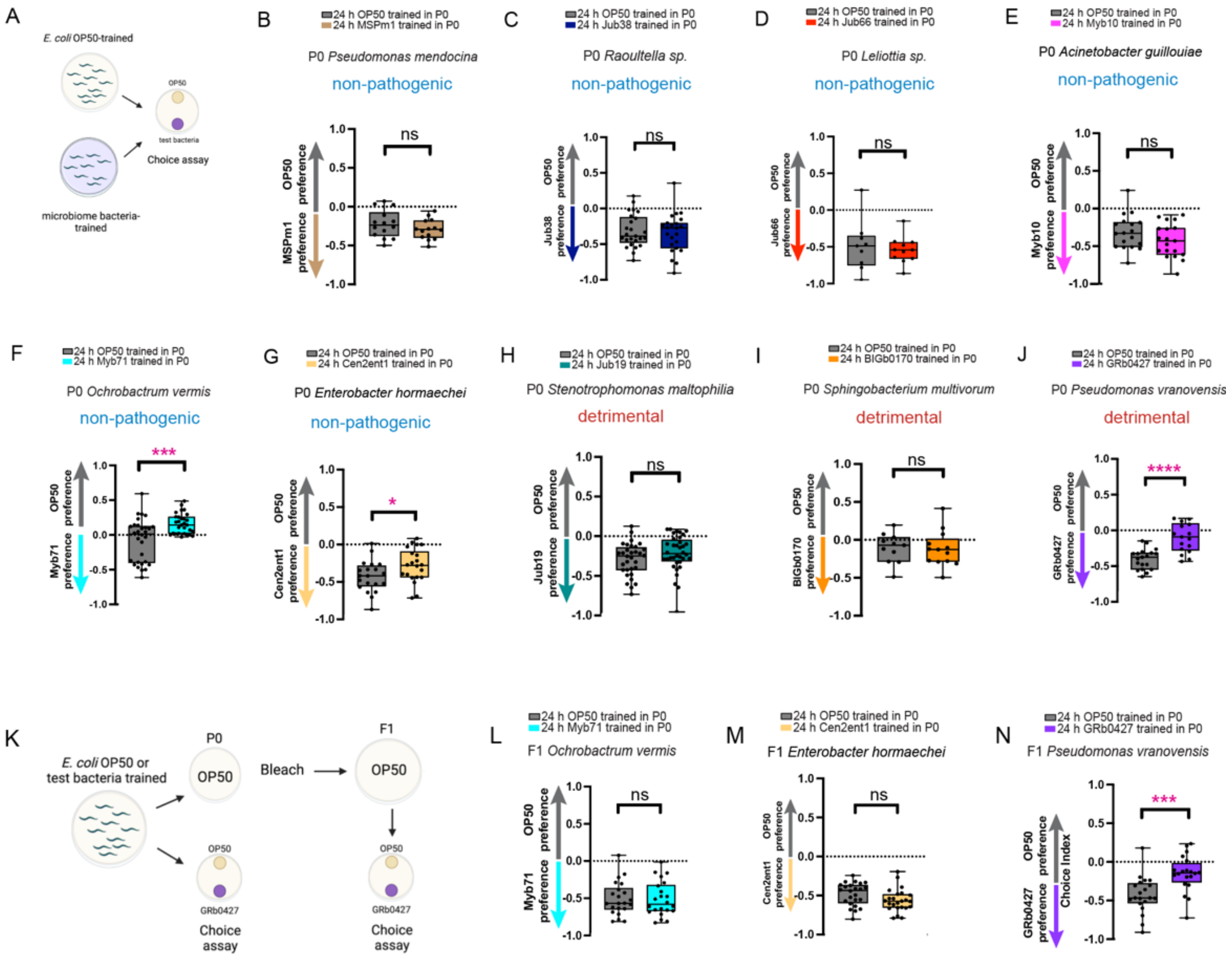


2) Need to explain what MACO-1 is early before introducing dependence on it in Fig. 5. What is the protein, what is its function, why is it important for memory etc… Why is a microtubule binding protein at all important for transmitting memory? Is this protein binding to anything differently when exposed to a memory inducing bacteria than regular bacteria? Is it differentially modified? Some additional experimental reason for the

importance of this protein would greatly strengthen the paper. This didn't seem to be provided in last manuscript or this one and the readers are left wondering why this particular protein is important here.

Thank you for pointing this out. We have added more information about *maco-1* and its many roles in various *C. elegans* and mammalian behavior and neuronal function. However, we still do not understand exactly why *maco-1* is important for sRNA-regulated learned pathogen avoidance, only that it is in the pathway and lies upstream of *daf-7* expression, and we have added this comment. It is the topic for further study. The study of additional examples will allow us to determine whether *maco-1* is the only target of bacterial sRNA-induced avoidance, or if other targets exist.

Minor points
1) First sentence of summary "its" is not appropriate here.

OK
2) Page 5 duplicate citations of 47 and 50.

Thank you for catching that error! Now fixed.

3) In Figure 1 it would be helpful to put an indicator on the actual figure of which strain is pathogenic, which are beneficial and which are positive/neutral depending on context. Or at least group them as you introduced them in the text. It's also confusing to have multiple different nomenclature for the same thing (Jub10 and Stenotrophomonas maltophilia). Just pick one nomenclature and stick with that (I would suggest the scientific one rather than the jargony abbreviation which can be put in the methods).

This is a very helpful suggestion – thank you. (We often use the strain name because there are occasionally multiple strains for a species.)

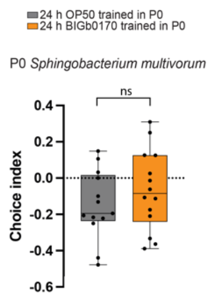


4) Do the other strains exhibit learning after longer exposure to the bacteria or is it only those 3 at any length of training? And do those other strains not have any small RNAs at all homologous to maco-1?

To address the reviewer's question, we repeated the training on one of the strains, *Sphingobacterium multivorum*, for 36 hrs, but still saw no effect (right). We have added these data to the paper. Thank you for the suggestion.

The fact that the other strains do not induce any F1 learning means that the P0 avoidance likely only induces previously-described non-sRNA (innate immunity) mechanisms, which is

outside of our interests here. We also have no information about the sRNAs made by any of the other strains – in fact, we had to sequence the sRNAs of *P. vranovensis* ourselves, as that information was not publicly available.

Our data suggest that only detrimental Pseudomonas strains (so far) have the ability to induce transgenerational epigenetic inheritance of learned avoidance.

5) Scale bars should be on every image not just on 1 of multiple images in a series (Fig 2A, C, E)

Fixed

6) What is the magnitude in increase in daf-7 GFP relative to exposure to PA14? Is this same magnitude, dramatically less, dramatically more? Positive control for Fig 2D

We have added PA14 treatment for comparison.

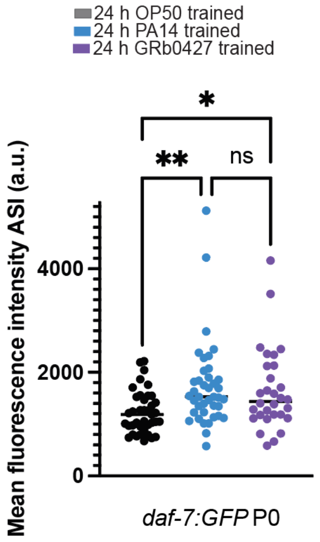


7) This sentence "This result is consistent with the daf-7p::gfp and irg-1p::gfp results (Figure 2E-F) suggesting that innate immunity pathways may not contribute significantly to C. elegans' avoidance of P. vranovensis, but rather that the major pathway of avoidance in P0s is through the same pathway as in F1-F4." Requires the reader to have extensive knowledge of your previous work. If you want to include this info need to summarize more briefly innate immune pathways etc from previous work and not assume reader has read your previous work.

Thank you, we will be sure to add more background information for this section.

8) I'm not sure what 3D provides that is different from 3C and same for 4E vs 4D. I'd say remove the second one as it is less informative. Or put to supplemental as they appear redundant.

OK

9) I got a bit confused as to what the difference between the IntReg is and the Pv1? I thought the IntReg was the maco-1 homology region but then in the next paragraph and figure you say it is Pv1??

Sorry for any confusion, which we will also clarify in the text. IntReg is a 347 nt chunk of the intergenic region that includes the 16nt match to *maco-1*; cloning this region when we first identified it in the *P. vranovensis* genome allowed us to test whether there could be an effect from this region. Once we had the sRNA sequencing data, we could then just work with the sRNA (Pv1).

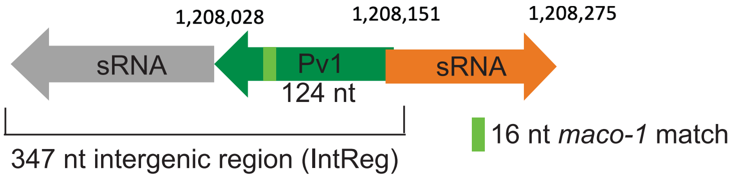


10) Figure 7D needs controls to show in that particular experiment choice is still working in your hands and that they weren't flukes. (preference to OP50). Same for 8C.

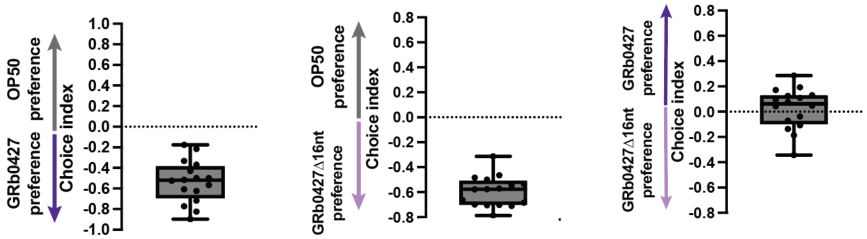


Shown are the controls done at the same time; we have added these back to the figures to show that the preferences are as expected.

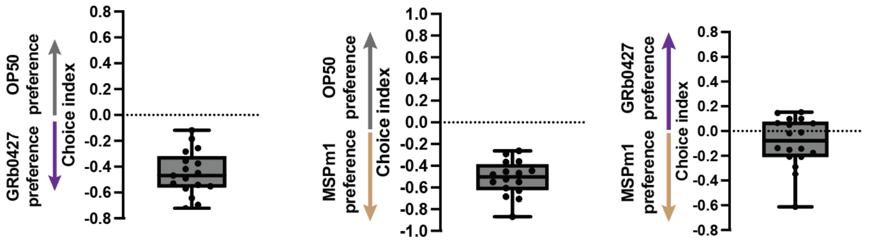


11) The temperature experiments in Figure 8a+b seem like an offshoot and not germane to major point of manuscript. Move them to supplementary or better to just remove completely.

It is clear from multiple reviewers' comments that we need to explain the logic here better. This part of the study is actually critical for the manuscript, as it addresses what the point of "forgetting" learned avoidance of a wild pathogen might be.

Because PA14 is a clinical isolate, it was important to ask if the same factors might affect transgenerational persistence. Our previous logic with PA14 was that temperature differences that affect pathogenesis might help explain why avoidance is terminated rather than hardwired. Therefore, we needed to test temperature effects on *P. vranovensis*. When

we found that temperature had no effect, we needed to explore other options to understand better why the avoidance effect might be terminated after a few generations. What could be the benefit of a return to naïve attraction to a pathogen? The most obvious option to us was that perhaps when avoiding the pathogen, the worms might also avoid a beneficial food source, and that could only happen if the food "smelled" the same to the worms as the pathogen. We found that this was indeed the case – training on *P. vranovensis* also caused avoidance of the beneficial (food) bacteria, *P. mendocina*. While of course this does not prove that learned avoidance of one wild pathogenic bacteria will cause avoidance of another, beneficial bacteria, our data do establish that such a situation could arise.
